# Detection of Gallbladder Disease Types Using a Feature Engineering-Based Developed CBIR System

**DOI:** 10.3390/diagnostics15050552

**Published:** 2025-02-25

**Authors:** Ahmet Bozdag, Muhammed Yildirim, Mucahit Karaduman, Hursit Burak Mutlu, Gulsah Karaduman, Aziz Aksoy

**Affiliations:** 1 Department of General Surgery, School of Medicine, Firat University, Elazığ 23119, Turkey; abozdag@firat.edu.tr; 2 Department of Computer Engineering, Malatya Turgut Ozal University, Malatya 44210, Turkey; burakmutlu44@gmail.com; 3 Department of Software Engineering, Malatya Turgut Ozal University, Malatya 44210, Turkey; mucahit.karaduman@ozal.edu.tr; 4 Department of Computer Engineering, Firat University, Elazığ 23119, Turkey; 5 Department of Bioengineering, Malatya Turgut Ozal University, Malatya 44200, Turkey; aziz.aksoy@ozal.edu.tr

**Keywords:** artificial intelligence, gallstone diseases, CBIR, carcinoma, cholecystitis

## Abstract

**Background/Objectives:** Early detection and diagnosis are important when treating gallbladder (GB) diseases. Poorer clinical outcomes and increased patient symptoms may result from any error or delay in diagnosis. Many signs and symptoms, especially those related to GB diseases with similar symptoms, may be unclear. Therefore, highly qualified medical professionals should interpret and understand ultrasound images. Considering that diagnosis via ultrasound imaging can be time- and labor-consuming, it may be challenging to finance and benefit from this service in remote locations. **Methods:** Today, artificial intelligence (AI) techniques ranging from machine learning (ML) to deep learning (DL), especially in large datasets, can help analysts using Content-Based Image Retrieval (CBIR) systems with the early diagnosis, treatment, and recognition of diseases, and then provide effective methods for a medical diagnosis. **Results:** The developed model is compared with two different textural and six different Convolutional Neural Network (CNN) models accepted in the literature—the developed model combines features obtained from three different pre-trained architectures for feature extraction. The cosine method was preferred as the similarity measurement metric. **Conclusions:** Our proposed CBIR model achieved successful results from six other different models. The AP value obtained in the proposed model is 0.94. This value shows that our CBIR-based model can be used to detect GB diseases.

## 1. Introduction

Gallbladder (GB) disease, a frequent pathology, must be diagnosed accurately and early on. The GB is an intraperitoneal organ located on the right lower surface of the liver. The gallbladder is located close to the duodenum, pancreas, and transverse colon. Anatomically, it is divided into three parts: the fundus and body, infundibulum, and neck. By holding and releasing bile produced by the liver, the gallbladder contributes significantly to the digestion process. Bile is a fluid produced by the liver that aids the digestive system and is discharged into the duodenum through the bile duct system for digestion and fat absorption [1]. Bile acids, phospholipids, lecithin, cholesterol, and bilirubin are the main constituents of bile.

**Figure 1:**
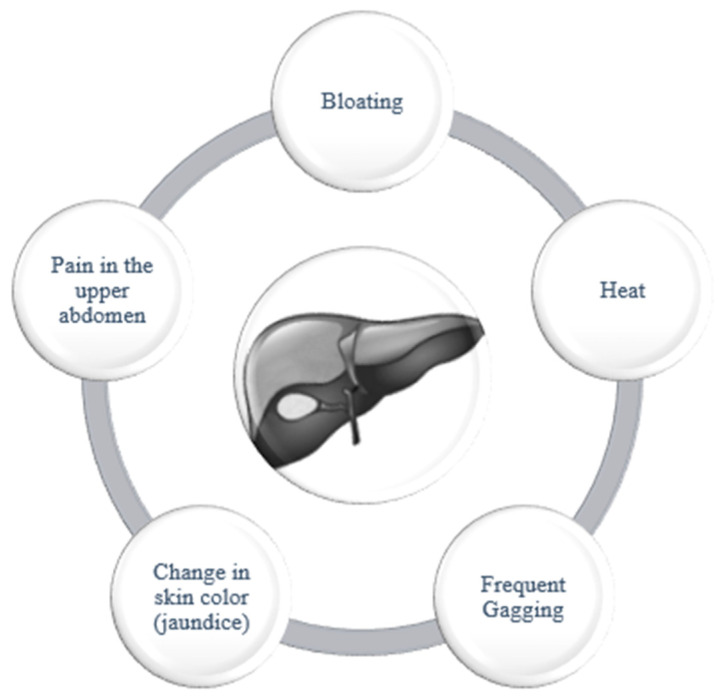
Main symptoms of gallbladder disease.

Cholelithiasis (gallstones) is the most prevalent GB condition, affecting about 10–15% of the adult population [2]. Nine pathological problems have been shown among the most common gallbladder diseases today. These include GB perforation, polyps and cholesterol crystals, gallbladder wall thickening, GB adenomyomatosis, gallstones, cholecystitis, gangrenous cholecystitis, carcinoma, and issues with the intraabdominal and retroperitoneum [3]. When the concentrations of the components that comprise bile surpass their solubility points, gallstones develop. Excessive cholesterol secretion relative to lecithin and bile salts causes cholesterol gallstone formation. However, it is known that gallstones are primarily due to genetic predisposition [4]. The risk also increases due to age, diabetes, and obesity. In Native Americans, prevalence rates of 30% in men and 60% in women have been shown [5]. The main symptoms of gallbladder disease are bloating, fever, frequent retching, change in skin color (jaundice), and pain in the upper abdomen, as shown in Figure 1 [6].

Before gallbladder surgery, the risk of choledocholithiasis should be assessed because gallbladder and common bile duct stones are frequently detected combined. Retrograde endoscopic cholangiography is used to remove stones from the common bile duct, and a cholecystectomy is performed if choledocholithiasis is verified by ultrasound, endoscopic ultrasound, or magnetic resonance cholangiography [7]. Gallstones are a major global health concern because, according to the recent research, they may put patients at risk for various illnesses, like cancer, cardiovascular disease, and even greater mortality [8,9,10]. Because of its aggressive nature and lack of effective treatment options, gallbladder carcinoma (GBC), the most prevalent malignant tumor of the biliary tract, has a terrible prognosis. The early detection of GBC is a great challenge for physicians, and most GBCs are incidentally detected during cholecystectomy procedures for gallstones [11]. Gallbladder polyps (GBPs) are elevated lesions of the gallbladder mucosa that extend into the gallbladder lumen. The prevalence of GBPs varies by region and ethnicity, ranging from 0.3% to 9.5%. Pathologically, GBPs can be separated into non-neoplastic polyps, such as gallbladder adenomyosis, cholesterol polyps, and inflammatory polyps, and neoplastic polyps, which are linked to adenomas and adenocarcinomas [12].

Because of the organ’s intricate anatomy and the variety of medical states that can affect it, diagnosing GB illnesses can be difficult. With the rapid growth of medical science and technology in recent years, artificial intelligence (AI) techniques have the potential to reduce human efforts significantly [13]. Based on ultrasound images (UIs), the AI field of deep learning (DL) is an instructive medical tomography technique that can aid in the early identification of GB disease. In order to assist medical personnel in identifying and categorizing different GB pathological anomalies, ML and deep learning (DL) approaches have recently demonstrated a notable capacity to analyze medical images [3]. By automating the analysis process and boosting diagnostic accuracy based on extensive datasets and sophisticated algorithms, these methods have the promising potential to improve GB diagnosis [14]. This study used a CBIR-based model to simultaneously detect nine gallbladder diseases and determine the disease type using UIs. The CBIR model is the search and analysis of different content-based image features.

CBIR is used to identify various schemas (modalities) in many images. Compared to a query image, CBIR lets clinicians retrieve pertinent images from an extensive database, minimizing time-consuming manual searches and aiding in diagnosis [15]. Based on a query image, CBIR seeks to identify related images from a big database. Numerous medical imaging domains are being actively researched in this discipline [16]. In addition to the deep learning architectures already mentioned, the suggested CBIR system offers interaction information to allow the user to choose which disease in the slice is being queried. CBIR can more effectively match images in this query for successful retrieval; that is, it can return more pertinent images. The CBIR system suggested in this paper can automatically produce pertinent image characteristics from well-annotated image datasets. It can also retrieve images to collect similar images and previous cases to obtain comprehensive information about the patient’s conditions.

Class consistency in the top-level images is a common way to evaluate a CBIR system’s quality. CBIR can improve the efficiency of time-consuming clinical workflow operations. The use of automated assistance systems, such as CBIR, in diagnosing numerous illnesses is growing in significance [16].

Images used in medicine are complicated. Due to the complex imaging data and the modest distinctions between disease states, automated techniques for objective lesion characterization are required. This approach can speed up radiology workflows and improve the overall quality of healthcare. Therefore, a CBIR-based model was developed, which produced successful results in GB diagnosis.

### 1.1. Releated Works

There are some studies in the literature for the detection of GB diseases. Unlike our study, deep learning-based methods were used in most of these studies. Lo et al. (2024) [17] collected a total of 2827 abdominal CT slices from computed tomography (CT) images to recognize the seven most relevant organs in the abdomen. DenseNet and transformer-based methods were preferred for the automatic detection of organs. Accuracy values in the range of 94–99% were achieved in the models used. Gupta et al. (2024) [18] compared the diagnostic performance of gallbladder cancer (GBC) with deep learning (DL)- based CNN architectures and skilled radiologists. The study included 565 patients, 334 of whom had gallstones and gallbladder diseases. When it came to detecting GBCs on the US CNN (0.836–0.945), Radiologist1 (0.733–0.891), and Radiologist2 (0.761–0.909), the DL-based method performed as well as or better than the expert radiologists. Zhou et al. (2024) [19] retrospectively trained a previously trained deep learning-based smartphone application to help diagnose biliary atresia from ultrasonographic gallbladder images using 3659 original sonographic gallbladder images and 51,226 smartphone photographs. A novice radiologist and an experienced pediatric radiologist also tested a new model. The new model’s diagnostic performance was more consistent and on par with that of seasoned pediatric radiologists. Yu et al. (2021) [20] recorded 89,000 abdominal US images from 2386 patients in the hospital database to detect and localize gallstones and cholecystitis with acceptable separation and speed. Using SSD-FPN-ResNet-50 and MobileNet V2 architectures, they determined accuracies of 0.92 and 0.94, respectively. Dadjouy and H. Sajedi (2024) [21] used the Faster R-CNN and YOLOv8 fusion method for gallbladder detection in ultrasound images to diagnose gallbladder cancer. Although the Faster R-CNN could estimate highly accurate bounding boxes, it also produced multiple bounding boxes that misidentified the background. In contrast, YOLO correctly estimated the location of the bounding boxes. This was achieved with 90.16% and 82.79% accuracies with Faster R-CNN and YOLOv8 fusion methods. Esen et al. (2024) [22] showed that the gradient boosting technique achieved the highest accuracy (85.42%) in predicting gallstones using a dataset consisting of Bioimpedance and the laboratory data of 319 individuals, 161 gallstone patients, and 158 healthy controls. Chattopadhyay et al. (2005) [23] detected gallbladder abnormalities with 92.3% accuracy from ultrasound scan (USS) images obtained from 751 cholecystectomy patients. Pang et al. (2019) [24] determined the diagnosis of cholelithiasis with 90.8% accuracy in the analysis of 1300 CT images of cholelithiasis patients with the CNN architecture (MobileNetV1, SSD, YOLOv2, and original SSD (with VGG-16)). Jang et al. (2021) [25] analyzed 1039 endoscopic ultrasound (EUS) images in patients with gallbladder polypoid lesions using the CNN architecture ResNet50. The rates were 57.9%, 96.5%, 77.8%, 91.6%, and 89.8% for EUS-AI in the differential diagnosis of neoplastic and non-neoplastic GB polyps. Veena et al. (2022) [26] analyzed the data from computerized tomography (CT) images of gallbladder diseases with SSD-efficient-Det, Faster R-CNN, and Mask R-CNNs models. They achieved 0.938 accuracy in the classification with Mask R-CNN architecture. In the study by Song et al. (2019) [27], the dataset included 5350 images from 726 patients who segmented CT images of gallbladder stones. In this study, the researchers achieved 91.68% success using DC-GAN and CNN architecture.

### 1.2. Contribution and Novelty

- It is seen that CNN-based architectures are primarily used in the literature to detect GB diseases. CNN-based architectures cannot produce successful results, especially in studies where the number of classes and data are high, and the training of the architectures takes a very long time in studies where large datasets are used.
- Considering that the dataset used in the study carried out for the detection of GB diseases has nine classes, we believe that this dataset is suitable for CBIR systems.
- Considering that the early detection of GB diseases is vital, it is very important to be able to detect these diseases at an early stage with computer-aided models.
- A new CBIR system has been developed to detect GB diseases early and highlight the success of CBIR systems in detecting GB diseases.
- In the developed system, feature extraction from different architectures and similarity measurement metrics were tested. As a result, features obtained from three different models were concatenated. At this stage, different features of the same image were used together.
- The results were also obtained with different CNN architectures and textural-based features to test the model’s performance. Different metrics were used to test the performance of the architectures.
- As a result, the proposed CBIR-based model obtained the most successful results in the detection of GB diseases.

### 1.3. Organization of Paper

In the rest of the article, the structure of the dataset used in the study, methods, similarity measurement metrics, feature extraction techniques, and the developed model are presented. Then, the experimental results, discussion, and conclusion sections are presented.

## 2. Materials and Methods

### 2.1. Datasets of Ultrasound Images of Gallbladder Diseases

Ultrasound images of the gallbladder organ taken from inside the digestive system made up the dataset. JPEG images of the digestive system were included in this dataset. Siemens Acuson X700, Philips Affiniti 70, Philips CX50, and Canon Viamo c100 ultrasound machines were used to create the images [28,29].

### 2.2. Data Processing and Data Collection

To maintain uniformity throughout the dataset, every image was enlarged to 900 × 1200 pixels with a 600 px horizontal resolution and 600 px vertical resolution, even though the original images had 450 × 600 pixels, a 3:4 aspect ratio, and 150 px horizontal and 150 px vertical resolutions. In total, 10,692 IU images from 1782 patients were used (Table 1) [29].

**Figure 2:**
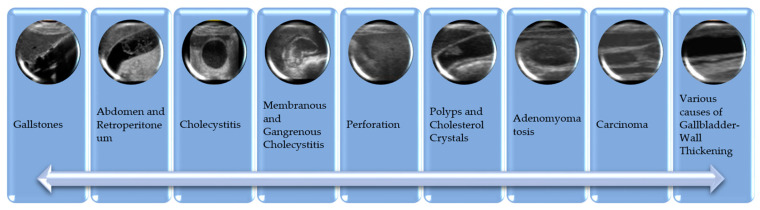
Gallbladder disease pathology IU images.

Sample images from the dataset are presented in Figure 2.

### 2.3. CBIR System Developed for the Detection of Gallbladder Disease Types

**Figure 3:**
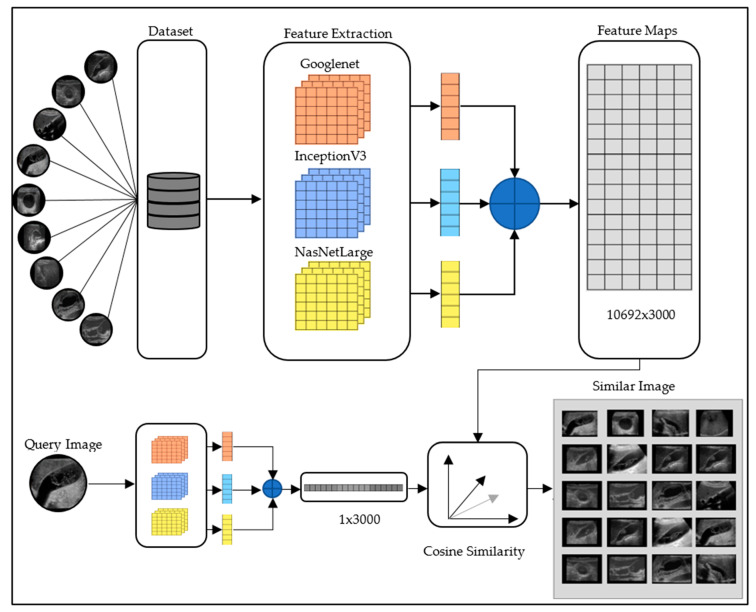
Developed CBIR system.

The feature map of the images related to the proposed model was extracted. This process produced a feature vector of 10,692 × 1000 corresponding to 10,692 images in the dataset. The proposed model combined feature maps extracted from three different models, aiming to work with different features of the same image. Finally, the combined feature maps were adopted as a feature extraction model in the developed CBIR system. The diagram of the suggested CBIR system is presented in Figure 3.

Image retrieval was conducted using the feature maps generated by the CBIR-based systems. In the proposed CBIR system, the feature map of the query image was extracted using the suggested model, and its performance was compared with other CNN architecture- and textural-based methods. The comparison was performed using cosines similarity measurement techniques, and the evaluation was carried out by analyzing the precision–recall (P-R) curve using an 11-point interpolated retrieval curve. In CNN architectures, Googlenet, InceptionV3, NasNetLarge, DenseNet201, and LBP and HOG architectures in machine learning classifiers were compared with the proposed model. The proposed model produced more successful results compared to the models used in this study.

### 2.4. Cosine Similarity Measure

The cosine similarity calculated for two vectors is the ratio of the calculation obtained from the product of the cosine angle of these two vectors. When the cosine similarity value of two vectors was 1, it was decided that they are similar. Equation (1) was used for the cosine similarity calculation [30].

(1) $$\mathrm{Cos}\alpha =\frac{A\cdot B}{\left|A\right|\cdot \left|B\right|}=\frac{\sum_{i=1}^{n}A_{i}\cdot B_{i}}{\sqrt{\sum_{i=1}^{n}{{(A}_{i})}^{2}}\cdot \sqrt{\sum_{i=1}^{n}{{(B}_{i})}^{2}}}$$

**Figure 4:**
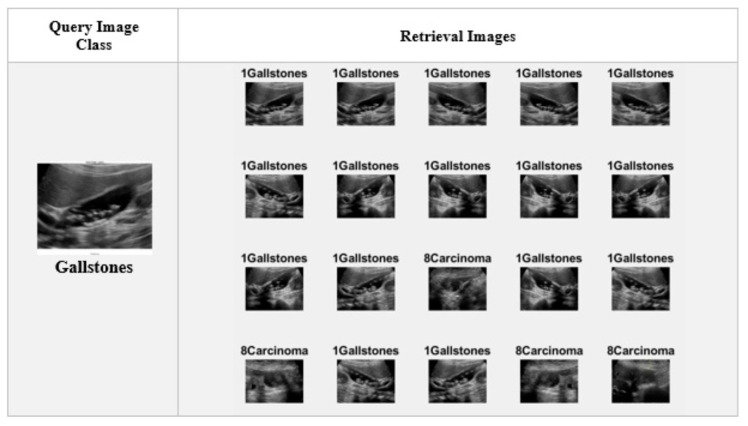
Examples of the queried image with the proposed CBIR systems.

In Equation (1), A represents the weight of each feature of vector A, and B represents the weight of each feature in vector B. According to the cosine similarity rule, the smaller the angle for comparing two vectors, the greater the degree of similarity. Twenty related images in the dataset were accessed by the suggested CBIR model, as seen in Figure 4. From the dataset’s classes, one image was chosen at random, and the 20 related images that were retrieved are displayed in order. The images accessed in the correct class and those accessed in the wrong are indicated with class labels.

Figure 4 shows 20 images taken from the dataset by querying a random image belonging to the gallstones class. As a result of the query, images 1–12, 14, 15, 17, and 18 are in the actual class, while images 13, 16, 19, and 20 are in different classes.

## 3. Application Results

The MATLAB 2024b program was used to obtain the findings of this research on a computer running Windows 10, 64 bit, equipped with an Intel i7 processor and 32 GB of RAM.

The order in which the images are accessed is crucial in content-based image access. Standard P-R graphs with 11 points are used for the evaluations. A P-R graph is provided for 20 randomly selected images in each class in the CBIR system that we created. The P-R graph employed the cosine approach, one of the techniques for measuring similarity.

The interpolated 11-point sequential access was assessed in this study using the average precession value and the P-R curve. The data collection consists of nine classes. The average P-R curve was obtained by querying each of the nine classes’ images separately. Twenty images were retrieved from the CBIR system after each class’s images were queried independently. By analyzing the images accessed and queried in each class, the average P-R curve was produced. A total of 10,692 images in the dataset were queried after the P-R curves of nine classes were assessed independently. The average P-R curve of the 20 images retrieved in each query was then computed and assessed.

**Figure 5:**
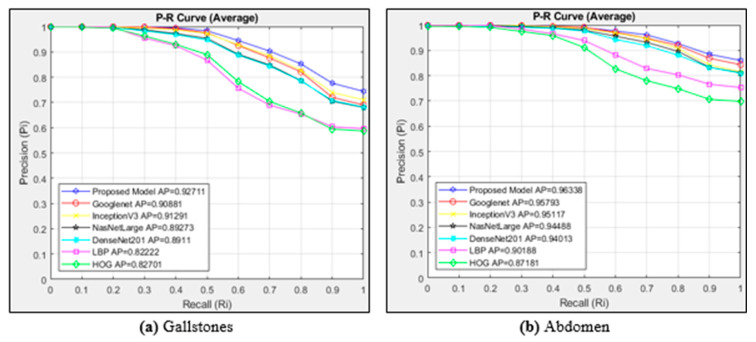
Average P-R curves for the classes of (**a**) gallstones and (**b**) abdomen.

Figure 5a shows the P-R curve for the gallstones class of six different architectures and the proposed model. The cosine method is used as the distance measurement metric. Since there are 1326 images in the gallstones class, 26,520 images are accessed. Figure 5b plots the curves of six different architectures and the proposed model for the images in the abdomen class. The cosine method is used as the distance measurement metric. Since there are 1170 images in the abdomen class, 23,400 images are accessed.

In Figure 5a, the P-R curves are calculated for the gallstones class using the cosine similarity measurement method for different models and the proposed model. While the proposed model achieved the highest AP value in the gallstones class, the least successful architecture was LBP. In the gallstones class, CNN-based methods achieved more successful results than textural-based methods. The AP value of the proposed model was 0.92711. The proposed model was successful in all cases in the recall {0…1} range in the gallstones class compared to the other six models. The models showed a high performance in cases up to Ri 0.2. When Figure 5b is examined, it can be seen that the proposed model shows the best performance in the abdomen class with an AP value of 0.96338. The textural-based HOG method performed the worst in the abdomen class, with an AP value of 0.87181. CNN architectures also produced more successful results than textural-based architectures in the abdomen class. Googlenet, InceptionV3, NasNetLarge, and DenseNet201 architectures achieved similar performances in the abdomen class.

**Figure 6:**
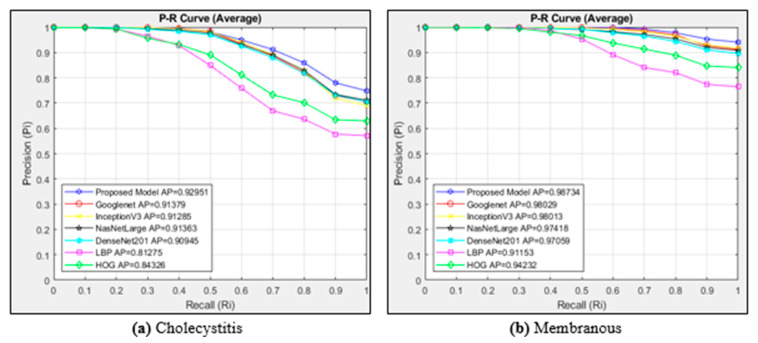
Average P-R curves for the classes of (**a**) cholecystitis and (**b**) membranous.

Figure 6a shows the P-R curves obtained for the cholecystitis class using the cosine distance measurement metric in different models. Since there are 1146 images in the cholecystitis class, 22,920 images are accessed. Figure 6b plots the curves of six different architectures and the proposed model for the images in the membranous class. The cosine method is used as the distance measurement metric. Since there are 1224 images in the membranous class, 24,480 images are accessed.

In Figure 6a, the P-R curves are calculated for the cholecystitis class using the cosine similarity measurement method for different models and the proposed model. While the proposed model achieved the highest AP value in the cholecystitis class, the least successful architecture was LBP. In the cholecystitis class, CNN-based methods achieved more successful results than textural-based methods. The AP value of the proposed model was 0.92951. The proposed model was successful in all cases in the recall {0...1} range in the cholecystitis class compared to the other six models. The models showed high performances in cases up to Ri 0.2. When Figure 6b is examined, it can be seen that the proposed model shows the best performance in the membranous class with an AP value of 0.98734. The textural-based LBP method performed the worst in the membranous class, with an AP value of 0.91153. CNN architectures also produced more successful results than textural-based architectures in the membranous class. NasNetLarge, DenseNet201, Googlenet, InceptionV3, HOG, and LBP architectures followed the proposed model.

**Figure 7:**
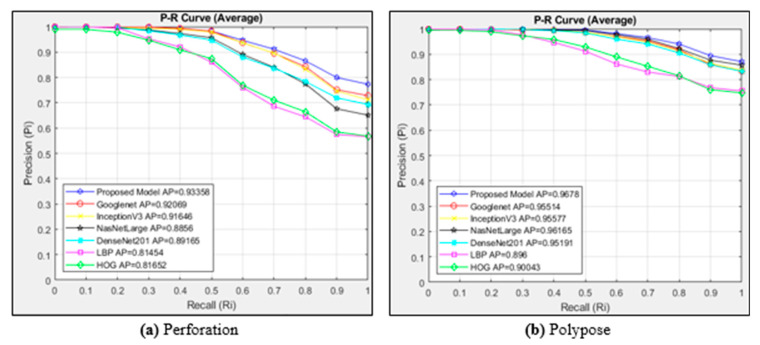
Average P-R curves for the classes of (**a**) perforation and (**b**) polypose.

The P-R curve for the perforation class images is shown in Figure 7a. This figure plots the curves of six different architectures and the proposed model. Since there are 1062 images in the perforation class, 21,240 images are accessed. Figure 7b plots the curves of six different architectures and the proposed model for the images in the polypose class. The cosine method is used as the distance measurement metric. Since there are 1020 images in the polypose class, 20,400 images are accessed.

Figure 7a plots P-R curves for the perforation class using the cosine distance measurement method. The proposed model obtained the highest AP value in the perforation class. The AP value of the proposed model is 0.93358. The least successful model in the perforation class is LBP, with an AP value of 0.81454. The highest AP value after the proposed model was achieved with the Googlenet architecture. The proposed model achieved a performance close to 1 in the range of 0–0.4. In Figure 7b, the most successful model in the polypose class was the proposed model with an AP value of 0.9678. Similar values were obtained in the other four CNN architectures used for comparison purposes. Textural-based models achieved lower AP values. In the proposed model, Ri decreased after the value of 0.5. It remained close to 1 until this interval.

**Figure 8:**
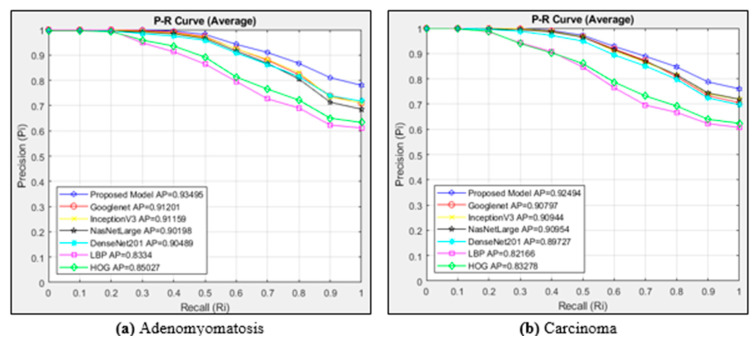
Average P-R curves for the classes of (**a**) adenomyomatosis and (**b**) carcinoma.

The P-R curve for the adenomyomatosis class is shown in Figure 8a. The curves of six different architectures and the suggested model are plotted in this figure. A total of 23,280 images are viewed since the adenomyomatosis class contains 1164 images. The curves of six different architectures and the suggested model for the images in the carcinoma class are plotted in Figure 8b. The metric for measuring distance is the cosine technique. A total of 31,800 images are viewed since the carcinoma class contains 1590 images.

According to the P-R curve in Figure 8a, the most successful model among the models used for image retrieval in the adenomyomatosis class is the proposed model with an AP value of 0.93495. Googlenet and InceptionV3 followed the proposed model with very close AP values. The proposed model was more successful than the other models in the Ri 0–1 range. The least successful models in the adenomyomatosis class were textural-based models. LBP was the least successful model in this class, with an AP value of 0.8334. When Figure 8b is examined, among the models used for image access in the carcinoma class, the most successful is the proposed model with an AP value of 0.92494. Googlenet, NasNetLarge, and InceptionV3 followed the proposed model with very close AP values. The proposed model was more successful than the other models in the Ri 0–1 range. These models performed in a close range at Ri 0–0.3. The least successful models in the carcinoma class were textural-based models. LBP was the least successful model in this class, with an AP value of 0.82166.

**Figure 9:**
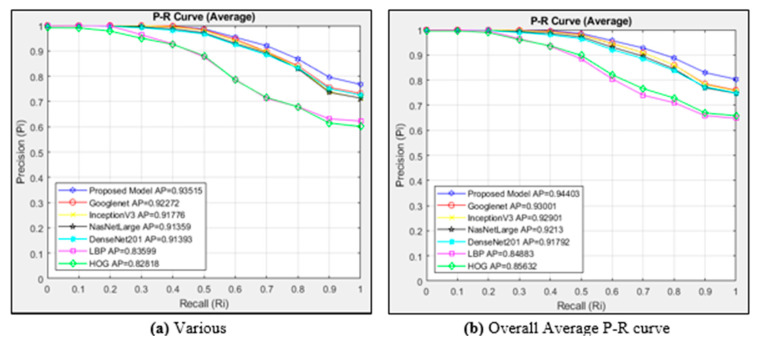
Average P-R curves for the classes of (**a**) various and (**b**) overall average P-R curves.

The P-R curves obtained using the cosine metric for the various class of six different architectures and the proposed model are presented in Figure 9a. A total of 19,800 images are viewed since the various classes contain 990 images. The P-R curves of all images in the nine classes in the dataset are shown in Figure 9b. This figure plots the curves of six different architectures and the proposed model. When the general performance curve is plotted, the cosine similarity measurement metric is used as the distance measurement metric. Since there are 10,692 images in the dataset, 213,840 images were accessed. In all steps, 20 images were accessed for each image.

Figure 9a compares the models and the proposed model using the cosine similarity measurement method. Our suggested model performed the best in the CBIR system, with AP = 0.93515 in the various classes of images. With an AP of 0.82818, the HOG architecture performed the worst in the various class average. The suggested model outperforms the existing models in image access in every scenario within the recall {0..1} range, according to an analysis of the P-R graph. Compared to other structures, the HOG architecture exhibits very little success and is similar to the LBP architecture.

When the images in the classes gallstones, abdomen and retroperitoneum, cholecystitis, membranous and gangrenous cholecystitis, perforation, polyps and cholesterol crystals, adenomyomatosis, carcinoma, and various causes of gallbladder wall thickening were evaluated separately in our CBIR system, our proposed model achieved success in all classes. InceptionV3, NasNetLarge, Googlenet, DenseNet201, LBP, and HOG architectures were less successful than our proposed model in all classes.

As in all classes, our suggested model performs better in our suggested CBIR system than alternative models when the overall average of all classes is analyzed in Figure 9b. Our suggested model’s InceptionV3, NasNetLarge, and Googlenet architectures perform similarly when employed alone, but less successfully when it comes to image retrieval. In terms of overall success, the LBP architecture performed the worst.

In Figure 9b, our proposed model, which consists of the combination of Googlenet + InceptionV3 + NasNetLarge architectures, is more successful in similar image retrieval with the feature extraction method and the proposed CBIR system using the cosine similarity measurement metric, where AP = 0.94403. Class-based performance measurement metrics are presented in Table 2.

## 4. Discussion

GB disease is a common pathology that requires accurate and early diagnosis for optimal medical treatment. The early diagnosis and appropriate treatment of gallbladder diseases are important for preventing complications. A healthy lifestyle, a balanced diet, and regular health check-ups can reduce the risk of developing these diseases. A delay in the diagnosis process or misdiagnosis can cause deterioration in the patient’s condition. Today, the use of AI, ML, and DL techniques in predicting disease progression, identifying abnormalities, and estimating mortality rates associated with GB diseases has increased rapidly in the last decade [3,31]. In the proposed CBIR system, 10,692 US images obtained from gallbladder diseases were analyzed and compared with textural-based models LBP and HOG, CNN architectures DenseNet201, Googlenet, InceptionV3, and NasNetLarge models, and disease verification was achieved with an average of 94.4%. Some studies on the subject and the proposed CBIR-based model are presented in Table 3.

Due to the cost of the training process of CNN-based models, CBIR systems are very important in studies with large datasets and many classes. When Table 2 is examined, it can be seen that the proposed CBIR-based model produces successful results. It is also possible to mention some limitations of the study. One of the limitations of this study is the use of data obtained from a single center. Another limitation is that the proposed CBIR system has been tested on a single dataset. The model may need to be tested on other datasets to prove its effectiveness. In subsequent studies, it is possible to include data and experts from different centers and obtain more general and successful results.

## 5. Conclusions

This study proposed a CBIR-based classification model for diagnosing gallbladder diseases using UI datasets. By comparing feature extraction methods, including machine learning architectures like LBP and HOG, and CNN architectures, such as DenseNet201, Googlenet, InceptionV3, and NasNetLarge, the model demonstrated a superior performance with the cosine similarity metric. The evaluation included class-specific and overall dataset analyses using interpolated 11-point PR curves and AP calculations for performance measurements. The proposed model achieved the best performance across nine gallbladder disease classes and the entire dataset. Specifically, the cosine similarity-based CBIR system yielded notable AP scores, such as 0.92711 for gallstones, 0.96338 for abdomen and retroperitoneum, and 0.98734 for membranous and gangrenous cholecystitis. Overall, the model demonstrated an impressive AP of 0.94403 for the entire dataset, outperforming other architectures in retrieval accuracy. In conclusion, the proposed CBIR system with the cosine similarity effectively enhances image retrieval and classification for gallbladder diseases, showcasing its potential for clinical applications and advancing diagnostic methodologies.

## Figures and Tables

**Table 1:** Gallbladder disease type dataset details.

Gallbladder Disease Type	Female	Male	Number of Patients	Number of Images
Gallstones	137	84	221	1326
Intraabdominal and retroperitoneum problems	110	85	195	1170
Cholecystitis	102	89	191	1146
Membranous and gangrenous cholecystitis	109	95	204	1224
Perforation	95	82	177	1062
Polyps and cholesterol crystals	99	71	170	1020
Adenomyomatosis	108	86	194	1164
Carcinoma	155	110	265	1590
Various causes of GB wall thickening	92	73	165	990
Total	1.007	775	1.782	10.692

**Table 2:** Class-based performance metrics of the models (AP).

Diseases	Proposed Model	Googlenet	InceptionV3	NasNetLarge	DenseNet201	LBP	HOG
Gallstones	0.92711	0.90881	0.91291	0.89273	0.8911	0.82222	0.82701
Abdomen and Retroperitoneum	0.96338	0.95793	0.95117	0.94488	0.94013	0.90188	0.87181
Cholecystitis	0.92951	0.91379	0.91285	0.91363	0.90945	0.81275	0.84326
Membranous and Gangrenous Cholecystitis	0.98734	0.98029	0.98013	0.97418	0.97059	0.91153	0.94232
Perforation	0.93358	0.92069	0.91646	0.8856	0.89165	0.81454	0.81652
Polyps and Cholesterol Crystals	0.9678	0.95514	0.95577	0.96165	0.95191	0.896	0.90043
Adenomyomatosis	0.93495	0.91201	0.91159	0.90198	0.90489	0.8334	0.85027
Carcinoma	0.92494	0.90797	0.90944	0.90954	0.89727	0.82166	0.83278
Various Causes of Gallbladder Wall Thickening	0.93515	0.92272	0.91776	0.91359	0.91393	0.83599	0.82818
General Average	0.94403	0.93001	0.92901	0.9213	0.91792	0.84883	0.85632

**Table 3:** Literature review.

Paper	Year	Model/Method/Architecture	Dataset	Images	Accuracy (%)
Dadjouy and Sajedi, 2024 [21]	2024	Faster R-CNN and YOLOv8	Gallbladder Cancer Ultrasound (GBCU)	The GBCU dataset includes 1255 ultrasound images from 218 patients	90.16%, 82.79%
Chattopadhyay et al., 2005 [23]	2005	Ultrasound scan (USS)	Polypoidal lesions in the gall bladder (PLG): ultrasound scan (USS)	751 cholecystectomies	Gallbladder abnormality (specificity) 92.3%
Esen et al., 2024 [22]	2024	RF, MLP, LR, NB, DT, KNN, GB, AdaBoost, and XGBoost	Bioimpedance and laboratory data	319 samples, 161 gallstone patients, and 158 healthy controls	85.42%
Yu et al., 2021 [20]	2021	SSD-FPN-ResNet-50 and MobileNet V2	89,000 abdominal US images taken from 2386 patients	Abdominal US images (>89,000)	0.92 and 0.94
Pang et al., 2019 [24]	2019	MobileNetV1, SSD, YOLOv2, and original SSD (with VGG-16)	CT images of 100 patients with cholelithiasis	A total of 1300 CT images of cholelithiasis	90.8%
Jang et al., 2021 [25]	2021	ResNet50	Endoscopic ultrasound (EUS):	1039 EUS images: polypoid lesions of the gallbladder (GB)	57.9%, 96.5%, 77.8%, 91.6%, 89.8%
Veena et al., 2022 [26]	2022	SSD-efficient-Det, Faster R-CNN, and Mask R-CNN models	computerized tomography (CT) images	60	Mask R-CNN has a value of 0.938
Song et al., 2019 [27]	2019	DC-GAN and CNN	Segmenting CT images of gallstones	The dataset includes 5350 images from 726 patients	91.68%
Proposed Model	2025	CBIR-based system	Gallbladder diseases	10.692 IU images	94.4%

## Data Availability

A publicly available dataset was used in this article.
